# N-terminal pro-brain natriuretic peptide measurements in hemodynamically significant patent ductus arteriosus in preterm infants

**DOI:** 10.12669/pjms.323.9690

**Published:** 2016

**Authors:** Shehab Ahmed Alenazi

**Affiliations:** 1 Dr. Shehab Ahmed Alenazi, MBBS, MD. Fellow of Saudi Pediatrics Board, Department of Pediatrics, Collage of Medicine, Northern Border University, Arar, Kingdom of Saudi Arabia

**Keywords:** HsPDA, NT-proBNP, Preterm infants

## Abstract

**Objectives:**

Evaluate the role of NT-proBNP levels in Preterm neonates suffering from PDA and used as a screening tool for predicting HsPDA and guiding physicians to consider early echocardiographic evaluation.

**Methods:**

This is a monocentric prospective blind study which was conducted at Arar Central Hospital, Ar’ar, Saudi Arabia, during the period between Jan 2014 to June 2014. Thirty-three (33) preterm infants born at less than 31 weeks of gestation or weighing less than 1200 g at birth infants were initially enrolled during a 6-month period. Blood samples were collected along with routine blood tests on days 1, 2, 3, and 7 of life for NT-proBNP analysis. Two echocardiographies were systematically performed on day two of life to ascertain about the status of Ductus Arteriosus.

**Results:**

The Plasma NT-proBNP levels were high on day one of life and decline from day three to day seven of life except in those infants with significant hsPDA. Plasma NT–proNBP levels on day 2 of infants in the HsPDA group were significantly higher (<0.001) than those in non-HsPDA group. Echocardiogram parameters indicates the significant difference (p<0.002) in Left Atrial and Aortic ratio (LA/AO), Interventricular septum thickness (P<0.03), Left ventricular posterior wall thickness (p<0.05), diastole PDA gradient (p<0.005) between HsPDA and non-HsPDA.

**Conclusions:**

Plasma NT-proBNP level peaked during the first few days after birth and declined rapidly within a week. Therefore, its level may have a role as a screening tool to predict HsPDA and provide more information regarding its spontaneous closure or otherwise.

## INTRODUCTION

Patent ductus arteriosus (PDA) is a common problem in premature infants born before 32 weeks.^1^ It refers to the persistent patency of the ductus arteriosus after birth^2^ characterized by a ductal shunt that diverts part of the aortic blood flow towards the pulmonary circulation, resulting in pulmonary vascular overload and decreased blood flow to systemic organs.^3^ In most full term infants, the ductus arteriosus closes during the first 48 hours after birth.^4^ However, in many small preterm infants the ductus remains open allowing an increasing systemic-to-pulmonary shunt as the pulmonary vascular resistance falls due to the elevated sensitivity of the ductal tissue to the dilating effects of prostaglandins and low sensitivity to the effects of oxygen.^5^ As a result, the ductus of preterm infants can remain open for many days, and although it may constrict initially, it frequently reopens^6^ resulting in, the decreased in the systemic blood flow, while the pulmonary blood flow is increased.^7^ This is labelled as hemodynamically significant PDA (HsPDA).

In HsPDA there is significant shunting of blood from the systemic to the pulmonary circuit resulting in increased pulmonary blood flow with reduction in effective systemic blood flow.^8^ Therefore, HsPDA is considered to be associated with various comorbid illnesses in premature infants,^9^ observed in more than 30% of premature infants with gestational ages of <32 weeks.^10^ Early diagnosis of hsPDA allows early treatment and probable reduction of morbidity in preterm infants.^11^ It is of vital importance to recognize and properly treat hemodynamically significant patent ductus arteriosus (hsPDA) in preterm infants.^12^ Because physical examination may be unreliable for determining the presence and magnitude of PDA in premature infants, echocardiography is used to document PDA shunting.^13^ However, routine echocardiography for the evaluation of PDA in premature infants has certain disadvantages, such as high cost, discomfort, disruption of the neonatal environment, and limited availability in some centers.^14^ Moreover, the hemodynamic effect of HsPDA may be difficult to determine even by using echocardiography, and the clinical course cannot be predicted reliably.^15^

Therefore a biomarker like NT-proBNP is a useful tool in diagnosis of PDA in premature neonates, especially in cases where Doppler echocardiography is not easily available^16^ as NT-proBNP concentration is significantly increased in infants with a PDA and correlates well with PDA diameter in the first three weeks of life.^17^ NT–proBNP is the inactive fragment of cardiac hormone B-type natriuretic peptide (BNP)^18^ which is secreted mainly in the ventricles in response to pressure or volume overload and increased wall stress.^19^ It is highly sensitive and specific indicator of hsPDA in low birth weight premature infants.^20^ Therefore, NT-proBNP levels may be a useful tool to identify neonates at risk of developing hsPDA in premature infants.^21^

The objective of this study was to evaluate the role of NT-proBNP levels in Preterm neonates suffering from PDA and to see whether it may be used as a screening tool for predicting HsPDA and guiding physicians to consider early echocardiographic evaluation.

## METHODS

### Study

This monocentric prospective blind study was conducted at Arar Central Hospital, Ar’ar, Saudi Arabia, during the period between Jan 2014 to June 2014.

### Subjects

Thirty-five (35) preterm infants were initially enrolled during a 6-month period (from Jan 2014 to June 2014). The data of two infants were excluded from analysis because the data were incomplete. Thirty one 33 infants (21 male, 12 female) were eligible for evaluation. Their mean GA was 28.6 (±2.6) weeks and the mean birth weight was 991 (±294) g.

### Inclusion criteria

The Preterm infants born at less than 31 weeks of gestation or weighing less than 1200 g at birth. The infants were enrolled within 24 hours of birth.

### Exclusion criteria

The Preterm infants with the presence of cardiac congenital anomaly other than PDA, life-threatening congenital malformation, severe asphyxia at birth, persistent pulmonary hypertension, and death within the first week of life.

### Methodology

We prospectively evaluated the infants for PDA within the first 24 h of life determine whether the patient is suffering from hsPDA or non-HsPDA diagnosis based on a large ductal flow with left to right shunt on color Doppler echocardiography.

1. Blood samples were collected along with routine blood tests on days 1, 2, 3, and 7 of life for NT-proBNP analysis.
2. Two echocardiographies were systematically performed on day two of life.

### NT-proBNP Laboratory Analysis

Blood collected for routine sampling in lithium–heparin tubes was transported to the laboratory at room temperature where it was centrifuged at 20009g for 6 minutes. Supernatant plasma was analyzed for NT-proBNP using the Roche Elecsys 2010 electrochemiluminescence immunoassay, following the manufacturer’s recommendations. In most referral hospitals in Saudi Arabia, these tests are frequently available and are carried out free of cost in these hospitals whenever the physicians feel, they send a request and tests are done.

### Echocardiographic measurements

Echocardiographies were performed using an 8–MHz probe (Philips CX 50). Conventional echocardiographic measurements were obtained in accordance with the guidelines of the American Society of Echocardiography.^22^ A two-dimensional, color Doppler, pulse-wave Doppler and M-mode echocardiogram was performed simultaneously. Echocardiographic parameters representing the degree of PDA shunting and volume load included PDA diameter detected by two dimensions and color Doppler, the left atrium to aortic root ratio (LA ⁄ Ao) detected by M-mode. The follow-up echocardiogram was performed on day two and whenever hsPDA was suspected. All echocardiographic findings would be blinded to the primary care team, unless other congenital heart defects were detected, which would exclude the infants from the study per the exclusion criteria.

### Diagnosis of hsPDA (Clinical criteria)

The definition of Echocardiographic hsPDA was based on the evidence from the previous report, and the diagnosis was made if at least two of the following criteria were met: (i) LA⁄Ao ratio ≥1.4, (ii) ductal diameter ≥1.5 mm and (iii) the percentage of the ratio of the TVI of the diastolic retrograde flow to the TVI of the systolic anterograde flow along the descending aorta ≥30%.^23^

According to Nuntnarumit et al; the Clinical hsPDA was defined as the presence of the ductal flow with predominant left to right shunt on color Doppler which measured at least 1.5mm on two-dimensional echocardiography plus at least two of the following signs, including heart murmur, persistent tachycardia (heart rate >160 beats/min), hyperactive precordium, bounding pulse, pulse pressure >25 mmHg, hepatomegaly, pulmonary hemorrhage (defined as blood or blood-stained fluid aspirated from the endotracheal tube in association with a respiratory deterioration and radiographic evidence of pulmonary hemorrhage), increasing respiratory support by 20% increase in oxygen supplementation or in pressure support and chest radiographic evidence of cardiomegaly or pulmonary congestion.^24^

### Statistical analysis

Statistical analysis was performed using Statistical Package for the Social Sciences (SPSS) version 15.0. Continuous variables are presented as mean values with standard deviation. P<0.05 is considered significant.

## RESULTS

Demographic and clinical parameters are listed in this table showing; mean±SD, Gestational age in weeks (wks), Birth weight in grams (gm), etc. Table-I.

**Table-I:** Demographic and clinical parameters.

*Characteristics*	*Total (*n*=33)*	*HsPDA (*n*=18)*	*Non-HsPDA (*n*=15)*	*p Value*
Gestational age (wks)*	28.6 (±2.6)	27.6 (±2.7)	29.0 (±2.4)	0.03
Birth weight (gm)*	991 (±294)	899 (±276)	1153 (±278)	0.003
Male gender (%)	21 (64)	13(62)	8 (38)	0.6
Caesarian section (%)	24 (73)	17 (71)	7 (29)	0.78
Apgar score 1 min†	6 (2-9)	6 (2-8)	6 (2-9)	0.02
Apgar score 5 min†	7 (4-9)	7 (5-8)	8 (4-9)	0.002

* Mean (±SD); †Median (range); P value by student t test.

Mean (± SD) of NT-proBNP levels in pmol ⁄ L on day1, 2, 3 and 7 of PDA are shown in Table-II. It indicates Plasma NT-proBNP levels were high on Day-1 of life and showed a decline from Day- 3 to Day-7 of life except in those infants with significant hsPDA. Plasma NT–proNBP levels on day 2 in the HsPDA group (n=18) were significantly higher than in non-HsPDA group (n=15). A cut-off NT–proBNP on day 2 of 7252 pmol/L offered the best predictive values for HsPDA. In non-HsPDA group, the plasma level of NT–proBNP was found to peak in the first two days of life (1286 pmol/L) and then declined during day 3–7 after birth. Plasma NT–proNBP levels on Day-2 of infants in the HsPDA group were significantly higher (<0.001) than those in non-HsPDA group.

**Table-II:** Mean (± SD) of NT-proBNP levels in pmol ⁄ L on day 1, 2, 3 and 7 of PDA.

*Days of PDA*	*HsPDA (*n*=18)*	*Non-HsPDA (*n*=15)*	*P value*
Day 1	2562 (± 98)	1118 (± 89)	<0.001
Day 2	7252 (± 125)	1286 (± 119)	<0.001
Day 3	7130 (± 147)	622 (± 44)	<0.001
Day 7	1588 (± 102)	156 (± 23)	<0.001

Echocardiogram parameters in the PDA infants, which indicates the significant difference (p<0.002) in Left Atrial and Aortic ratio (LA/AO), Interventricular septum thickness (P<0.03), Left ventricular posterior wall thickness (p<0.05), diastole PDA gradient (p<0.005) between HsPDA and non-HsPDA are shown in Table-III.

**Table-III:** Echocardiogram parameters on day 2.

*Parameters*	*HsPDA (*n*=18)*	*Non-HsPDA (*n*=15)*	*P value*
LA/AO*	1.45 (±0.24)	1.25 (±0.23)	0.002
Left ventricular diastolic diameter*	11.8 (±2.1)	12.4(±2.0)	0.26
Interventricular septum thickness*	3.0 (±0.6)	3.3 (±0.7)	0.03
Left ventricular posterior wall thickness*	2.0 (±0.5)	2.3 (±0.6)	0.05
Shortening fraction (%)*	37(±4)	36 (±4)	0.36
PDA gradient systole*	10.2 (±9.9)	18.0 (±12.0)	0.06
PDA gradient diastole*	4.3 (±5.1)	11.5 (±8.0)	0.005
Tricuspid regurgitation (mmHg)*	22.4 (±13.1)	12.4 (±7.8)	0.005

PI – Pulsatility index; SMA – Superior mesenteric artery;

* Mean (±SD); P value – By students t test.

## DISCUSSION

In this study we observed that the Plasma NT-proBNP levels were high on Day-1 of life and showed a decline from Day-3 to Day-7 of life except in those infants with significant hsPDA. Plasma NT–proNBP levels on day 2 in the HsPDA group (n=18) were significantly higher than in non-HsPDA group (n=15). In non-HsPDA group, the plasma level of NT–proBNP was found to peak in the first two days of life (1286 pmol/L) and then declined during day 3–7 after birth which is highly predictive of a ductus that will close spontaneously.^25^ Plasma NT–proBNP levels on Day- 2 of infants in the HsPDA group were significantly higher (<0.001) than those in non-HsPDA group. Thus our results are in agreement with Letshwiti et al. study.^19^ Previous studies like of Koch et al, showed that plasma concentration of NT-proBNP is high in the first few days after birth due to changes in circulatory volume leading to increased ventricular volume and pressure load.^26^

El-Khuffash et al. also reported a significantly high level of NT-proBNP at 2 day of life, not at Day-1, in preterm infants with HsPDA and a rapid decrease after successful PDA closure. 27 Farombi-Oghuvbu et al. studied serial plasma NT-proBNP levels on day 1, 3, 5 and 10 of life and found that the plasma NT-proBNP levels on day 3, not on day 1 or day 10, were significantly higher in infants with HsPDA.^28^ These findings as well as ours suggest that the best timing to measure NT-proBNP level for predicting HsPDA should be at day 2–3 of life. Recent studies have showed that the NT-proBNP level on Day-1 of life was affected by gestational age with the level being higher in the more premature infants.^29^

In Farombi-Oghuvbu et al study the high levels of NT-proBNP on day 10 were also found in infants with sepsis.^28^ Therefore, high levels of NT-proBNP should be interpreted with caution. In our study, no infants developed clinical sepsis during the first week of life; high levels of NT-proBNP are only reflection of PDA status. Our results regarding heart geometry in PDA infants are also in agreement with Occhipinti et al.^30^ as NT-proBNP plasma concentrations are related to ductal size and left atrial dilatation in preterm infants during the first days after birth. LA/Ao ratio, in our study, correlated with the plasma NT-proBNP level after adjusting for other related variables.

Another study of El-Khuffash et al. has also shown that NT-proBNP on day 3 significantly correlated with LA/Ao ratio, and PDA diameter.^31^ A low NT-proBNP level could therefore represent an independent marker of PDA spontaneous closure at a time when classical echographic signs are poorly predictive of spontaneous evolution of the PDA.^32^ The peak plasma NT-proBNP level on day 2 at 7252 pmol/ml offered the best predictive values for HsPDA. These results were similar to those of the recent study using NT-proBNP on day 3 as a marker for HsPDA in preterm infants.^33^ Therefore, the NT-proBNP level on day 2–3 may be used as a screening tool in preterm infants for predicting HsPDA and guiding physicians to consider early echocardiographic evaluation. NT-proBNP in conjunction with an echocardiography may identify preterm infants with PDA who are at risk for poor outcome and would benefit from targeted medical treatment of PDA.

## CONCLUSIONS

Plasma NT-proBNP level peaked during the first few days after birth and declined rapidly within a week. Significantly high levels of plasma NT-proBNP on Day-2 were observed in infants who developed HsPDA. Therefore, its level may have a role as a screening tool to predict HsPDA and provide more information regarding its spontaneous closure or otherwise.
